# A significant risk of metabolic dysfunction-associated fatty liver disease plus diabetes on subclinical atherosclerosis

**DOI:** 10.1371/journal.pone.0269265

**Published:** 2022-05-31

**Authors:** Rieko Bessho, Kazuhiro Kashiwagi, Akihiko Ikura, Karin Yamataka, Jun Inaishi, Hiromasa Takaishi, Takanori Kanai

**Affiliations:** 1 Center for Preventive Medicine, Keio University Hospital, Tokyo, Japan; 2 Hills Future Preventive Medicine and Wellness, Keio University, Tokyo, Japan; 3 Division of Gastroenterology and Hepatology, Department of Internal Medicine, Keio University, Tokyo, Japan; Michigan State University, UNITED STATES

## Abstract

**Background:**

This cross-sectional study aims to investigate the association between subclinical atherosclerosis and metabolic dysfunction-associated fatty liver disease (MAFLD) or non-alcoholic fatty liver disease (NAFLD), and a synergistic effect of diabetes mellitus (DM) and MAFLD on subclinical atherosclerosis.

**Methods:**

Of 977 subjects who underwent health checkups with coronary artery calcification (CAC), carotid intima-media thickness, and brachial-ankle pulse wave velocity (ba-PWV), 890 were included in this study. They were classified as MAFLD, NAFLD, or Neither-FLD, and MAFLD was further categorized into three groups by three metabolic disorders (obesity, lean with metabolic dysregulation, DM), according to its new definition: Obesity-MAFLD, Lean-MAFLD and DM-MAFLD.

**Results:**

In a multivariable analysis, MAFLD and NAFLD were significantly associated with subclinical atherosclerosis, except for an association between ba-PWV and NAFLD. MAFLD had higher odds for CAC than NAFLD (for CAC score > 100, odds ratio (OR) = 2.599, 95% confidence interval (CI) = 1.625–4.157; OR = 1.795, 95%CI = 1.145–2.814, respectively). In a sub-analysis, DM-MAFLD had higher odds for CAC (for CAC score > 100, OR = 5.833, 95%CI = 3.047–11.164) than the other groups of MAFLD, when compared to Neither FLD as a reference. Moreover, DM-MAFLD had a higher level of homeostasis model assessment of insulin resistance and high sensitive C-reactive protein, compared to the other groups of MAFLD.

**Conclusions:**

MAFLD was significantly associated with subclinical atherosclerosis in the general population. Additionally, DM-MAFLD could be a significant risk factor for cardiovascular disease through insulin resistance and low-grade inflammation and requires careful follow-up or appropriate intervention.

## Introduction

Non-alcoholic fatty liver disease (NAFLD) is strongly associated not only with cirrhosis and carcinoma, but also with metabolic syndrome and its related components, leading to high morbidity and mortality from liver-related and extrahepatic diseases [1, 2]. The most common cause of death in patients with NAFLD is cardiovascular disease (CVD), independent of other metabolic comorbidities. Several studies have shown that NAFLD is a significant risk factor for atherosclerosis in the coronary and carotid arteries [3–6]. Furthermore, NAFLD has been suggested as an independent predictor of coronary artery calcification (CAC) using CAC score (CACS), a marker of a substitute for coronary arterial plaque burden evaluated by cardiac computed tomography (CT) [7–14]. In addition, NAFLD was reported to be strongly associated with subclinical atherosclerosis, including elevated brachial-ankle pulse wave velocity (ba-PWV) and carotid intima-media thickness (IMT) [15].

Very recently, an international expert group recommended “metabolic dysfunction-associated fatty liver disease (MAFLD)” as a more propriate term to highlight the importance of the metabolic dysfunction risk for CVD [16]. The definition of MAFLD includes hepatic steatosis (> 5% liver fat) plus any one of the following three groups [16]: 1) overweight/obesity; 2) lean/normal weight with specific metabolic dysregulation; 3) DM. A subsequent study reported that MAFLD associated more strongly with CVD than NAFLD [17]. However, no studies have comprehensively investigated the correlation between asymptomatic atherosclerosis such as CAC, ba-PWV, or carotid IMT, and MAFLD or MAFLD with DM (DM-MAFLD). Thus, this cross-sectional study aims to investigate the correlation of MAFLD and subclinical atherosclerosis, and to further examine whether MAFLD and DM may have a synergistic effect on subclinical atherosclerosis.

## Materials and methods

### Study setting and population

This cross-sectional study was conducted as part of a comprehensive study of NAFLD, approved by the Institutional Review Board of Keio University Hospital (IRB No. 20170384). The need for informed consent was waived by the ethics committee, because this retrospective study is reporting medical records. We also have discussed whether all data were fully anonymized before we accessed them. The inclusion criteria for this study was a series of 977 individuals who evaluated subclinical atherosclerosis using cardiac CT scan, ba-PWV and carotid artery ultrasound, as part of a health checkup at the Center for Preventive Medicine, Keio University Hospital, between August 2012 and December 2018. The exclusion criteria were as follows: heart diseases such as chronic heart failure or arrhythmia (n = 85) and data missing (n = 2). Thus, 890 subjects were included in the final analysis of the study.

### Collection of medical data

The following data were retrieved from their medical records: Demographics, medical history, obesity-related factors including body mass index (BMI) and visceral adipose tissue (VAT), and blood test data. BMI was calculated as weight (kg) divided by the square of height (m^2^). To measure VAT, umbilicus level Fat CT was performed and then calculated with AZE Virtual Place software (AZE Inc., Tokyo, Japan). The blood test included: total cholesterol (TC), triglyceride (TG), low-density lipoprotein cholesterol (LDL-C), high-density lipoprotein cholesterol (HDL-C), fasting blood sugar (FBS), hemoglobin A1c (HbA1c), homeostatic model assessment-insulin resistance (HOMA-R), estimated glomerular filtration rate (eGFR) and high sensitive C-reactive protein (hs-CRP). If the value of hs-CRP was greater than 0.14, it was defined as elevated.

The following self-administered questioners were routinely used to assess the lifestyle of subjects who underwent our health checkups: (Q1) Have you ever smoked? (Ever smoking, +/-). (Q2) Are you a non-drinker, or how much and how often do you drink alcohol? (Q3) Are you usually exercising over more than 30 minutes with a little sweat at least 2 days per week? (Exercise, +/-).

### Assessment of subclinical atherosclerosis and fatty liver

CAC was detected with a VCT XTe-64 slice multidetector CT scanner (GE Healthcare, Tokyo, Japan) by using the standard scanning protocol [14]. The calcium content in each coronary artery was measured and summed, and the total CACS was determined using the method by Agatston [14, 18]. CACS were classified into one of two grades (0 versus > 0, or ≤ 100 versus > 100).

PWV between the brachial and ankle sites and IMT of common carotid artery were evaluated as previously reported [19]. PWV was measured by an automatic waveform analyzer (Colin Medical Technology Corporation; Komaki, Japan) and elevated arterial stiffness was defined as ba-PWV > 1400 cm/s. The maximum IMT was measured using a high-resolution Logiq S8 system (GE Healthcare; Tokyo, Japan) and was determined to be elevated if it was 1.1mm or greater.

Fatty liver was diagnosed by the following ultrasound (US) findings: liver brightness, echo contrast between the hepatic and renal parenchyma, vascular burring and deep attenuation [20]. Our experienced sonographers performed these US examinations, followed by confirmation by the radiologist.

### NAFLD, and MAFLD and their grouping

NAFLD subjects were defined as those with (1) the presence of FL by US (2) daily alcohol consumption ≤ 30g for males and ≤ 20g for females (3) the absence of positive HBs antigen or HCV antibody [1]. MAFLD was diagnosed, according to the criteria [16], as those with FLD by US plus any one of the following three groups: 1) Obesity-MAFLD, overweight/obesity (≥ 23kg/m^2^); 2) Lean-MAFLD, lean/normal weight (≺ 23kg/m^2^) with specific metabolic dysregulation; 3) DM-MAFLD, type 2 DM. Metabolic dysregulation was defined as the presence of at least two metabolic conditions as follows: (1) waist circumference ≥ 90 cm in males and 80 cm in females, (2) blood pressure ≥ 130 mmHg for systolic, ≥ 85 mmHg for diastolic or specific drug treatment, (3) TG ≥ 150 mg/dl or specific drug treatment, (4) HDL-C ≺ 40 mg/dl for males and ≺ 50 mg/dl for females or specific drug treatment, (5) prediabetes (FBS 100–125 mg/dl or HbA1c 5.7–6.4%) (6) HOMA-R ≥ 2.5, and (7) CRP > 2 mg/L. Thus, MAFLD was subdivided into three groups: Obesity-MAFLD, Lean-MAFLD and DM-MAFLD.

### Evaluation of hepatic fibrosis by non-invasive hepatic fibrosis marker

To assess the association between hepatic fibrosis and each group of MAFLD, the fibrosis-4 index (FIB-4) was calculated as follows: FIB-4 = [age (years) x AST (IU/L)] / [platelet count (x 10^9^/L) x ALT (IU/L)^1/2^]. The cutoff points were chosen as 1.3 to divide low fibrosis and moderate-high fibrosis [21].

### Statistical analysis

For continuous data, mean values were expressed with standard deviation (SD), and statistical differences between two groups were determined using the *t*-test or Mann-Whitney U in the univariate analyses. For categorical data, numbers were presented with percentage, and statistical differences were determined using the chi-square tests. Then, a binary regression analysis was used to analyze the correlation between the dichotomous outcome (CAC score = 0 versus > 0, or > 100 versus ≤ 100) and MAFLD　or NAFLD. Two models were presented with progressive adjustments by covariates, which were chosen for clinical importance as well as statistical significance, including age, sex, hypertension, dyslipidemia, smoking status, physical activity and eGFR in this multivariable regression model. The presence of DM was not included in these models to avoid collinearity, since it was used to define DM-MAFLD. A sensitivity analysis was carried out to confirm the robustness of the associations by comparing using two types of controls. All statistical analyses were performed using SPSS software version 24 (SPSS, Inc., Chicago, Ill). All *p*-values less than 0.05 were considered statistically significant.

## Results

### Clinical characteristics of study population

**Fig 1:**
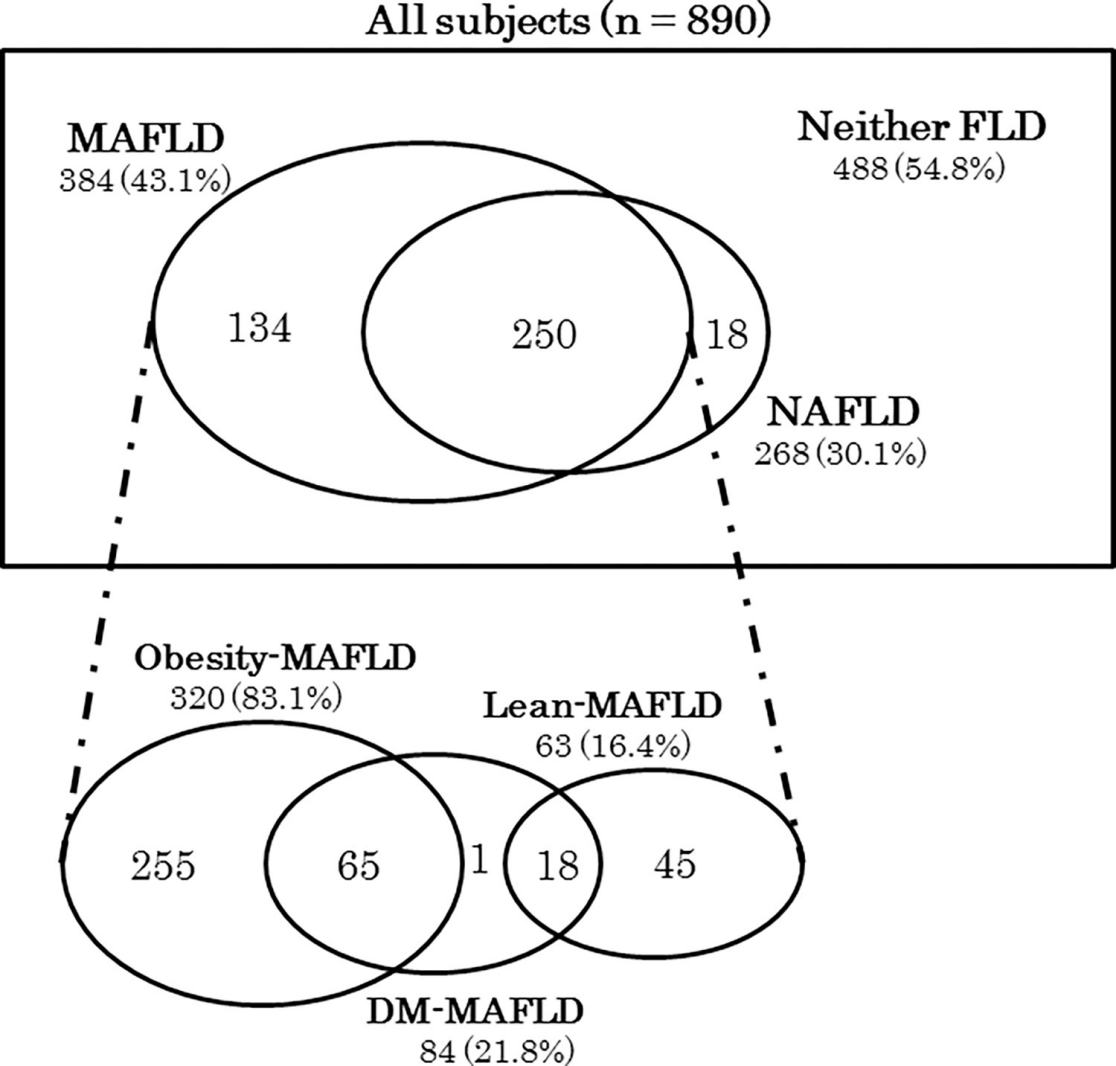
(Upper) The proportion of MAFLD and NAFLD according to the two definitions in the study population. (Lower) The proportion of each of the MAFLD groups. MAFLD, metabolic dysfunction-associated fatty liver disease; NAFLD, non-alcoholic fatty liver disease; DM, diabetes mellitus.

The mean age of 890 subjects was 60.2 ± 12.3 years and 598 were males (67.2%). **Fig 1** shows that MAFLD subjects were 384 (43.1%), which included 320 of Obesity-MAFLD, 63 of Lean-MAFLD and 84 of DM-MAFLD, whereas the prevalence of NAFLD subjects was 30.1%. Two hundred and fifty subjects (28.1%) belonged to both FLD. **Table 1.** demonstrates that MAFLD subjects were significantly associated with metabolic abnormalities and subclinical atherosclerosis, compared to those without (MAFLD-).

**Table 1:** Clinical characteristic by presence of MAFLD.

Characteristics	MAFLD+	MAFLD-	*P*
	384 (43.1)	506 (56.9)	
Age (years)	59.8 ± 11.0	60.6 ± 13.2	0.341
Male	308 (80.2)	290 (57.3)	**0.000**
Body mass index (kg/m^2^)	25.9 ± 3.7	21.8 ± 2.7	**0.000**
VAT (cm^2^)	133.9 ± 52.0	73.2 ± 39.0	**0.000**
**Questioners**			
Ever smoking	225 (58.6)	228 (45.1)	**0.000**
Exercise	136 (35.4)	199 (39.3)	0.233
Hypertension	175 (45.6)	156 (30.7)	**0.000**
Diabetes mellitus	84 (21.9)	42 (8.3)	**0.000**
Dyslipidemia	138 (35.9)	90 (17.7)	**0.000**
**Blood test**			
Total cholesterol (mg/dL)	203.4 ± 36.1	209.2 ± 35.5	**0.017**
LDL-C (mg/dL)	117.6 ± 30.3	115.5 ± 28.7	0.291
HDL-C (mg/dL)	50.6 ± 11.8	63.4 ± 15.9	**0.000**
Triglycerides (mg/dL)	147.0 ± 97.2	89.5 ± 45.0	**0.000**
Albumin (g/dl)	4.35 ± 0.29	4.27 ± 0.27	**0.000**
Platelet (x10^4^/ul)	22.3 ± 5.4	22.0 ± 5.4	0.294
Fasting blood sugar (mg/dL)	114.7 ± 23.3	102.9 ± 17.3	**0.000**
Hemoglobin A1c (%)	5.96 ± 0.68	5.66 ± 0.55	**0.000**
HOMA-R	2.4 ± 2.4	1.1 ± 0.8	**0.000**
Aspartate transaminase (U/L)	26.9 ± 13.5	23.2 ± 17.5	**0.000**
Alanine transaminase (U/L)	29.4 ± 18.9	20.5 ± 40.4	**0.000**
γ-GTP (U/L)	51.8 ± 68.4	33.9 ± 74.7	**0.000**
Elevated hs-CRP	73 (19.0)	50 (9.9)	**0.000**
**Subclinical atherosclerosis**			
CACS > 0	189 (49.2)	161 (31.7)	**0.000**
CACS > 100	78 (20.3)	44 (8.7)	**0.000**
ba-PWV > 1400 (cm/s)	200 (52.1)	227 (28.2)	**0.033**
c-IMT ≥ 1.1 (mm)	122 (31.8)	100 (19.7)	**0.000**

MAFLD, metabolic dysfunction-associated fatty liver disease; VAT, visceral adiposetissue; LDL-C, low-density lipoprotein cholesterol; HDL-C, high-density lipoproteincholesterol; HOMA-R, homeostasis model assessment of insulin resistance; γGTP,gamma-glutamyl transferase; hs-CRP; high sensitive C-reactive protein; CACS, coronary artery calcification score; ba-PWV, brachial ankle pulse wave velocity; c-IMT, carotid intima media thickness.

### The association of subclinical atherosclerosis with presence of MAFLD or NAFLD

As shown in **Table 2**, MAFLD and NAFLD were significantly associated with CACS, respectively, compared to MAFLD- or NAFLD- as a reference: for CACS > 0, odds ratio (OR) = 1.821, 95% confidence interval (CI) = 1.331–2.492; OR = 1.825, 95% CI = 1.320–2.524; for CACS > 100, OR = 2.599, 95% CI = 1.625–4.157; OR = 1.795, 95% CI = 1.145–2.814, respectively. MAFLD was also significantly associated with elevated ba-PWV and carotid IMT (OR = 1.562, 95% CI = 1.128–2.161; OR = 1.823, 95% CI = 1.287–2.580), whereas NAFLD was correlated only with the latter (OR = 1.999, 95% CI = 1.407–2.840).

**Table 2:** The association of subclinical atherosclerosis with presence of MAFLD or NAFLD.

Subclinical atherosclerosis		Model1	*P*	Model2	*P*
CACS > 0	MAFLD	1.980(1.455–2.694)	0.000	1.821(1.331–2.492)	0.000
	NAFLD	1.780(1.293–2.452)	0.000	1.825(1.320–2.524)	0.000
CACS > 100	MAFLD	3.201(2.036–5.034)	0.000	2.599(1.625–4.157)	0.000
	NAFLD	1.974(1.280–3.044)	0.002	1.795(1.145–2.814)	0.011
ba-PWV > 1400	MAFLD	1.786(1.302–2.449)	0.000	1.562(1.128–2.161)	0.007
	NAFLD		0.167		0.091
c-IMT ≥ 1.1	MAFLD	1.976(1.408–2.773)	0.000	1.823(1.287–2.580)	0.001
	NAFLD	2.076(1.472–2.927)	0.000	1.999(1.407–2.840)	0.000

MAFLD, metabolic dysfunction-associated fatty liver disease; NAFLD, non-alcoholic fatty liver disease; CACS, coronary artery calcification score; ba-PWV, brachial ankle pulse wave velocity; c-IMT, carotid intima media thickness.

Model 1: adjusted for age, sex. Model 2: adjusted items in Model 1 plus for hypertension, dyslipidemia, estimated glomerular filtration rate, ever smoking, and exercise.

### Sub-analysis of subclinical atherosclerosis in four groups with combination of MAFLD and/or DM

**Fig 2:**
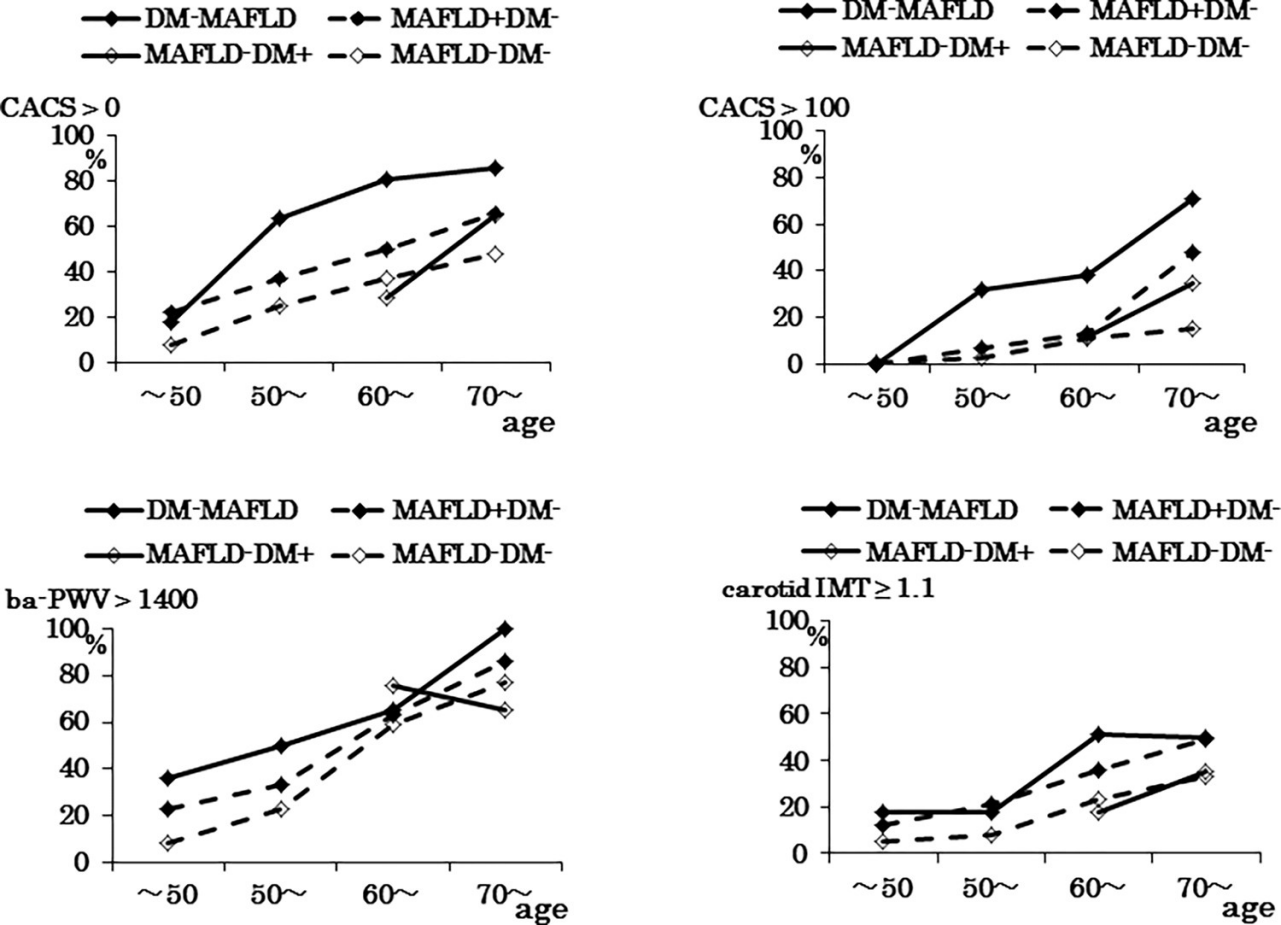
(Upper) The percentage of CACS > 0 and > 100. (Lower) The percentage of ba-PWV > 1400 cm/s and carotid IMT ≥ 1.1 mm, based on the combination of MAFLD and/or DM by age (under 50, 50s, 60s, 70 and over). DM-MAFLD, MAFLD with DM; MAFLD+DM-, MAFLD without DM; MAFLD-DM+, no MAFLD and DM; MAFLD-DM-, no MAFLD and no DM. MAFLD, metabolic dysfunction-associated fatty liver disease; DM, diabetes mellitus; CACS, coronary artery calcification score; ba-PWV, brachial ankle pulse wave velocity; IMT, Intima media thickness.

**Fig 2** shows the percentage of subclinical atherosclerosis in four groups, based on combination of MAFLD and/or DM by age (under 50, 50s, 60s, 70 and over): (1) no MAFLD and no DM (MAFLD-DM-, n = 464); (2) no MAFLD and DM (MAFLD-DM+, n = 42); (3) MAFLD without DM (MAFLD+DM-, n = 300); (4) MAFLD with DM (DM-MAFLD, n = 84). The risk-positive percentage of DM-MAFLD was higher than that of MAFLD+DM- across all ages in all risk indicators of subclinical atherosclerosis, and the latter was higher than that of MAFLD-DM-. The multivariable analysis demonstrated that DM-MAFLD had higher odds for subclinical atherosclerosis than MAFLD+DM- (for CACS > 0, OR = 3.913, 95% CI = 2.249–6.810 vs OR = 1.546, 95% CI = 1.102–2.171; for CACS > 100, OR = 7.218, 95% CI = 3.784–13.767 vs OR = 1.990, 95% CI = 1.163–3.404; for c-IMT ≥ 1.1, OR = 2.231, 95% CI = 1.287–3.868 vs OR = 1.704, 95% CI = 1.164–2.495, respectively), except for ba-PWV, when MAFLD-DM- was a reference (**Table 3**).

**Table 3:** The association of subclinical atherosclerosis with combination of MAFLD and/or DM.

Subclinical atherosclerosis		Model1	*P*	Model2	*P*
CACS > 0	MAFLD-DM-	1.00(reference)		1.00(reference)	
	MAFLD-DM+		0.884		0.884
	MAFLD+DM-	1.625(1.163–2.271)	0.004	1.546(1.102–2.171)	0.012
	DM-MAFLD	3.913(2.249–6.810)	0.000	3.913(2.249–6.810)	0.000
CACS > 100	MAFLD-DM-	1.00(reference)		1.00(reference)	
	MAFLD-DM+		0.175		0.175
	MAFLD+DM-	2.464(1.468–4.135)	0.001	1.990(1.163–3.404)	0.012
	DM-MAFLD	7.218(3.784–13.767)	0.000	7.218(3.784–13.767)	0.000
ba-PWV > 1400	MAFLD-DM-	1.00(reference)		1.00(reference)	
	MAFLD-DM+		0.858		0.681
	MAFLD+DM-	1.647(1.165–2.328)	0.005	1.490(1.046–2.124)	0.027
	DM-MAFLD	2.459(1.470–4.422)	0.001		0.075
c-IMT ≥ 1.1	MAFLD-DM-	1.00(reference)		1.00(reference)	
	MAFLD-DM+		0.652		0.652
	MAFLD+DM-	1.870(1.289–2.711)	0.001	1.704(1.164–2.495)	0.006
	DM-MAFLD	2.231(1.287–3.868)	0.004	2.231(1.287–3.868)	0.004

MAFLD, metabolic dysfunction-associated fatty liver disease; DM, diabetes CACS, coronary artery calcification score; ba-PWV, brachial ankle pulse wave velocity; c-IMT, carotid intima media thickness.

Model 1: adjusted for age, sex. Model 2: adjusted items in Model 1 plus for hypertension, dyslipidemia, estimated glomerular filtration rate, ever smoking, and exercise.

### Sub-analysis of subclinical atherosclerosis and hepatic fibrosis in the MAFLD groups

Lean-MAFLD and DM-MAFLD had a higher risk-positive percentage for subclinical atherosclerosis than Obesity-MAFLD, as shown in **Table 4,** and **S1 Fig**. When the Neither-FLD was a reference, the multivariable-adjusted odds [95% CI] for CACS > 0 and CACS > 100 were higher in DM-MAFLD (3.908 [2.258–6.764], and 5.833 [3.047–11.164]) than those in the other groups of MAFLD (**Table 5)**. Regarding the probability of moderate to high hepatic fibrosis estimated by the FIB-4 index, there seemed to be no significant difference among the three groups of MAFLD (**S2 Fig**).

**Table 4:** Clinical characteristic of each group of MAFLD.

Characteristics	Obesity-MAFLD	Lean-MAFLD	DM-MAFLD	*P
	320 (36.0)	63 (7.1)	84 (9.4)	
Age (years)	59.8 ± 11.0	64.8 ± 9.6	60.9 ± 9.4	0.000
Male	264 (82.5)	43 (68.3)	77 (91.7)	0.010
Ever smoking	189 (59.0)	35 (55.6)	65 (77.4)	0.606
Exercise	113 (35.3)	22 (34.9)	29 (34.5)	0.953
Non-drinker	80 (25.0)	19 (30.2)	13 (15.5)	0.393
Hypertension	150 (46.9)	25 (39.7)	54 (64.3)	0.285
Diabetes mellitus	106 (33.1)	18 (28.6)	84 (100.0)	0.146
Dyslipidemia	138 (35.9)	32 (50.8)	40 (47.6)	0.008
AST	27.7 ± 14.5	23.0 ± 5.6	30.6 ± 21.6	0.000
ALT	30.9 ± 20.0	24.5 ± 34.3	33.5 ± 28.3	0.000
γGTP	53.6 ± 72.2	43.0 ± 43.8	78.0 ± 128.7	0.261
HOMA-R	2.47 ± 1.98	2.13 ± 3.84	3.40 ± 4.13	0.305
Elevated hs-CRP	67 (20.9)	6 (9.5)	18 (21.4)	0.014
FIB-4 moderate-high	163 (50.9)	38 (60.3)	49 (58.3)	0.173
CACS > 0	153 (47.8)	36 (57.1)	58 (69.0)	0.176
CACS > 100	62 (19.4)	17 (27.0)	32 (38.1)	0.172
ba-PWV > 1400	166 (51.9)	41 (65.1)	53 (63.1)	0.058
c-IMT ≥ 1.1	90 (28.1)	32 (50.8)	32 (38.1)	0.000

MAFLD, metabolic dysfunction-associated fatty liver disease; AST, Aspartate transaminase; ALT, Alanine transaminase; γGTP, gamma-glutamyl transferase; HOMA-R, homeostasis model assessment of insulin resistance; hs-CRP; high sensitive C-reactive protein; CACS, coronary artery calcification score; ba-PWV, brachial ankle pulse wave velocity; c-IMT, carotid intima media thickness.

^*^P, *P*-value for Obesity-MAFLD vs Lean-MAFLD

**Table 5:** The association of subclinical atherosclerosis with presence of any one of MAFLD groups.

Subclinical atherosclerosis		Model1	*P*	Model2	*P*
CACS > 0	*Neither FLD	1.00(reference)		1.00(reference)	
	Obesity-MAFLD	1.888(1.361–2.621)	0.000	1.720(1.231–2.404)	0.001
	Lean-MAFLD	2.261(1.268–4.032)	0.006	2.261(1.268–4.032)	0.006
	DM-MAFLD	3.908(2.258–6.764)	0.000	3.908(2.258–6.764)	0.000
CACS > 100	Neither FLD	1.00(reference)		1.00(reference)	
	Obesity-MAFLD	3.011(1.863–4.868)	0.000	2.510(1.528–4.123)	0.000
	Lean-MAFLD	3.478(1.741–6.947)	0.000	2.682(1.300–5.532)	0.008
	DM-MAFLD	6.886(3.663–12.946)	0.000	5.833(3.047–11.164)	0.000
ba-PWV > 1400	Neither FLD	1.00(reference)		1.00(reference)	
	Obesity-MAFLD	1.856(1.323–2.603)	0.000	1.582(1.116–2.244)	0.010
	Lean-MAFLD		0.075		0.084
	DM-MAFLD	2.603(1.501–4.512)	0.001		0.104
c-IMT ≥ 1.1	Neither FLD	1.00(reference)		1.00(reference)	
	Obesity-MAFLD	1.714(1.187–2.475)	0.004	1.565(1.075–2.278)	0.019
	Lean-MAFLD	3.769(2.116–6.713)	0.000	3.769(2.116–6.713)	0.000
	DM-MAFLD	2.307(1.341–3.970)	0.003	2.307(1.341–3.970)	0.003

MAFLD, metabolic dysfunction-associated fatty liver disease; DM, diabetes CACS, coronary artery calcification score; ba-PWV, brachial ankle pulse wave velocity; c-IMT, carotid intima media thickness.

Model 1: adjusted for age, sex. Model 2: adjusted items in Model 1 plus for hypertension, dyslipidemia, estimated glomerular filtration rate, ever smoking, and exercise.

*Neither FLD was defined as neither MAFLD nor non-alcoholic fatty liver disease.

## Discussion

We found that MAFLD is significantly associated with CAC, elevated ba-PWV and carotid IMT, even after adjusted by age, sex, hypertension, dyslipidemia, eGFR, smoking, and exercise, whereas NAFLD had a significant association with the above subclinical atherosclerosis, except for ba-PWV. Moreover, MAFLD had higher odds of CACS > 100, in comparison with NAFLD. Thus, MAFLD could identify subclinical atherosclerosis better than NAFLD in the general population.

In this cross-sectional study, the prevalence of MAFLD determined by the new definition was 43.1%, compared to the prevalence of 30.1% for NAFLD, which is almost the same as some previous epidemiological studies [17, 22, 23]. In the comparison of MAFLD but without NAFLD (MAFLD only) to NAFLD (S1 Table), the former was predominantly male, with a higher proportion of drinkers or smokers, and a higher frequency of metabolic disorders such as hypertension or diabetes. This difference is believed to be due to differences in the diagnostic criteria for MAFLD and NAFLD. The proportion of coexisting DM with MAFLD (i.e., DM-MAFLD) among MAFLD in this study was slightly higher than in previous reports [17, 24] (21.8% vs 15.8–20.6%), whereas the number of non-MAFLD subjects with DM (i.e., MAFLD-DM+) was very small (n = 42).

The most common cause of death in patients with NAFLD is CVD [5, 6] and CACS has been shown to be a strong predictor of atherosclerotic CVD events; Compared to CACS of 0, CACS of 1–100 has four times higher risk of the event, and a CACS cutoff above 100 increases the risk of the event seven times [11, 13]. To date, three meta-analyses regarding CACS have reported that subjects with NAFLD had a significant association with subclinical coronary atherosclerosis compared to those without [9, 10, 25]. Specifically, a recent meta-analysis on diabetic population showed 2.2-fold increased risk for CVD in the NAFLD group, compared with the non-NAFLD group, suggesting that NAFLD and DM might have a synergistic effect on the risk of CVD [26]. However, as far as we know, no studies have comprehensively reported the relationship between subclinical atherosclerosis and MAFLD or its components, especially, DM-MAFLD. Therefore, in the sub-analysis, we compared DM-MAFLD to MAFLD-DM- or Neither FLD as a reference to examine the impact of DM on subclinical atherosclerosis. Importantly, DM-MAFLD had significantly higher odds for subclinical atherosclerosis compared to MAFLD-DM-, although MAFLD-DM+ did not significantly increase the risk of subclinical atherosclerosis. The small sample size belonging to the MAFLD-DM+ group may have affected this unexpected result. Also, subsequent sensitivity analysis demonstrated similar results: DM-MAFLD had higher odds for subclinical coronary atherosclerosis than the other groups of MAFLD, when compared to Neither FLD as a reference. Thus, the present study suggested that MAFLD and DM as comorbidities have a synergistic effect on the risk of subclinical atherosclerosis. On the other hand, Lean-MAFLD is composed of a population that is older and has more complications of hyperlipidemia compared to Obesity-MAFLD (Table 4). Lean-MAFLD had higher odds ratio of subclinical atherosclerosis than Obesity-MAFLD except ba-PWV (Table 5). Therefore, the location and extent of ectopic fat accumulation, such as fatty liver and epicardial adipose tissue, may contribute to the pathogenesis of subclinical atherosclerosis rather than obesity as defined by BMI.

Our study also suggests that there seemed to be no significant difference in the probability of moderate to high hepatic fibrosis among the three groups of MAFLD, although DM-MAFLD might have a slightly high probability of moderate to high fibrosis in subjects under the age of 60, compared to the other subgroups of MAFLD. Since most of DM-MAFLD was included in Obesity-MAFLD, the two groups were not independent. Therefore, it was difficult to statistically compare difference in the variables in FIB-4.

The underlying pathophysiological candidates linking the association between NAFLD and coronary atherosclerosis in an earlier stage include insulin resistance and low-grade hepatic and systematic inflammation [27, 28]. The main focus of the definition of MAFLD is metabolic dysfunction as a core element along with the accumulation of hepatic steatosis [16]. Serum level of AST, ALT and HOMA-R, and the proportion of elevated hs-CRP were significantly higher in subjects with MAFLD than those without. In addition, their level and the proportion were higher in the DM-MAFLD group than the other groups of MAFLD. Therefore, unlike the direct effects on the liver by fatty liver-induced inflammation and insulin resistance, their effects on coronary atherosclerosis might be amplified, especially due to inflammation and insulin resistance induced by metabolic dysfunction such as DM.

Our study has some limitations. First, this cross-sectional study cannot conclude a causal link between subclinical atherosclerosis and MAFLD. Second, the diagnosis of FLD was determined by US, not by more sensitive and specific modalities such as magnetic resonance imaging or US elastography. Third, the number of subjects in the MAFLD-DM+ group is very small, especially only five under the age of 60. Lastly, since our results are derived from Japanese health checkup data, the findings may not be generalized to other races. Thus, more longitudinal studies are needed to determine if there is a synergistic effect on subclinical atherosclerosis between MAFLD and DM.

## Conclusion

MAFLD, especially DM-MAFLD, was significantly associated with subclinical atherosclerosis in an asymptomatic general population. This study suggests that DM-MAFLD could be a significant risk factor for CVD through insulin resistance and low-grade inflammation, and requires careful follow-up or appropriate intervention.

## Supporting information

S1 Data (XLSX)

S1 Fig The risk-positive percentage of subclinical atherosclerosis in Neither FLD and the MAFLD groups. MAFLD, metabolic dysfunction-associated fatty liver disease; DM, diabetes mellitus; CACS, coronary artery calcification score; ba-PWV, brachial ankle pulse wave velocity; IMT, Intima media thickness.
(TIF)

S2 Fig The percentage of moderate-high hepatic fibrosis evaluated by the FIB-4 index in the MAFLD groups by age (under 50, 50s, 60s, 70 and over). MAFLD, metabolic dysfunction-associated fatty liver disease; DM, diabetes mellitus; FIB-4, fibrosis-4.
(TIF)

S1 Table Clinical characteristic of MAFLD only and NAFLD. MAFLD, metabolic dysfunction-associated fatty liver disease; VAT, visceral adipose tissue; LDL-C, low-density lipoprotein cholesterol; HDL-C, high-density lipoprotein cholesterol; HOMA-R, homeostasis model assessment of insulin resistance; γGTP, gamma-glutamyl transferase; hs-CRP; high sensitive C-reactive protein; CACS, coronary artery calcification score; ba-PWV, brachial ankle pulse wave velocity; IMT, Intima media thickness.
(DOCX)
