# Supplementary figures and images for: A significant risk of metabolic dysfunction-associated fatty liver disease plus diabetes on subclinical atherosclerosis
Source: PLoS One. 2022 May 31;17(5):e0269265. doi: 10.1371/journal.pone.0269265 (PMC9154100; doi:10.1371/journal.pone.0269265)

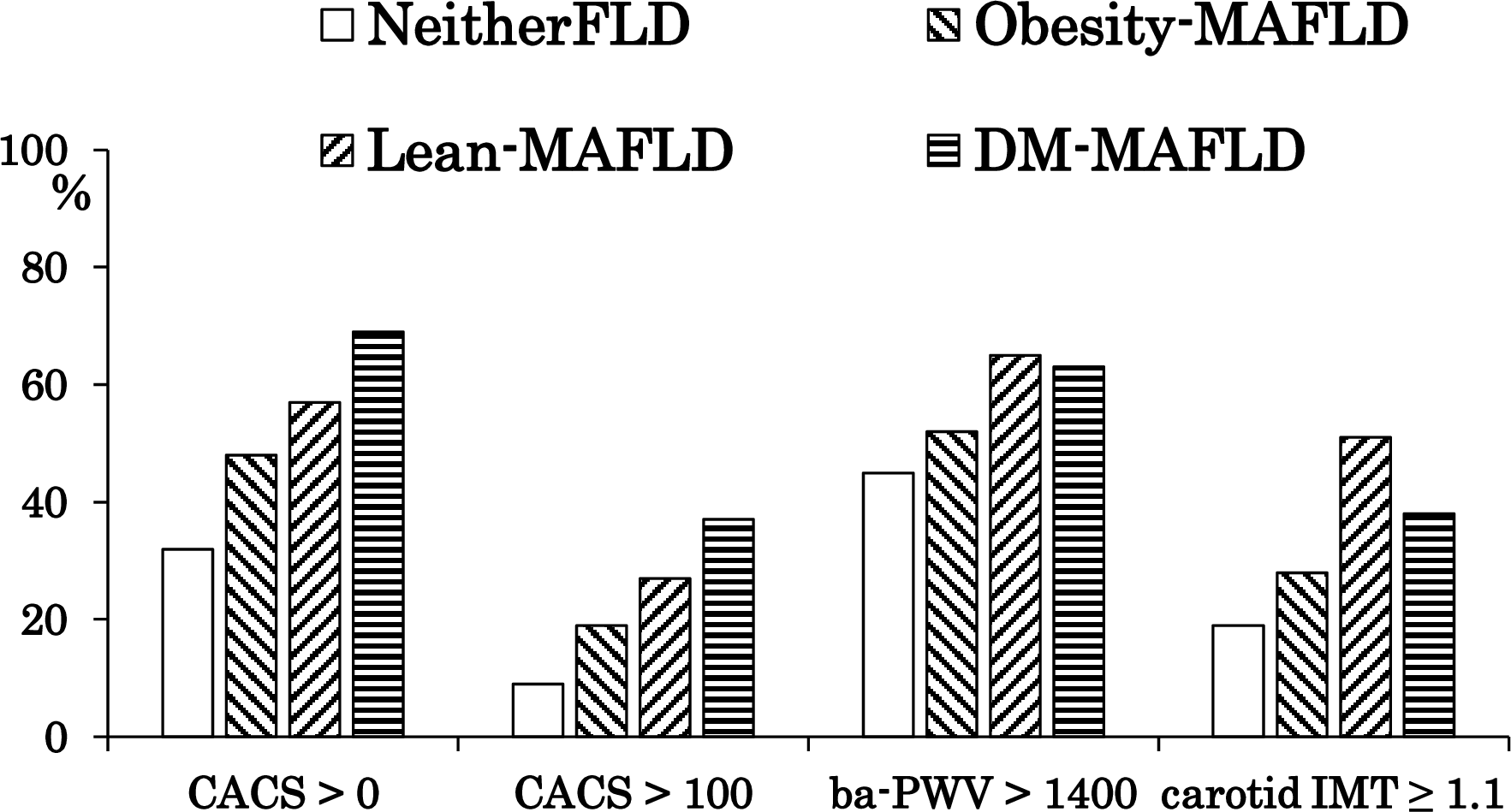

Supplement: S1 Fig — MAFLD, metabolic dysfunction-associated fatty liver disease; DM, diabetes mellitus; CACS, coronary artery calcification score; ba-PWV, brachial ankle pulse wave velocity; IMT, Intima media thickness. (TIF) [file pone.0269265.s002.tif]

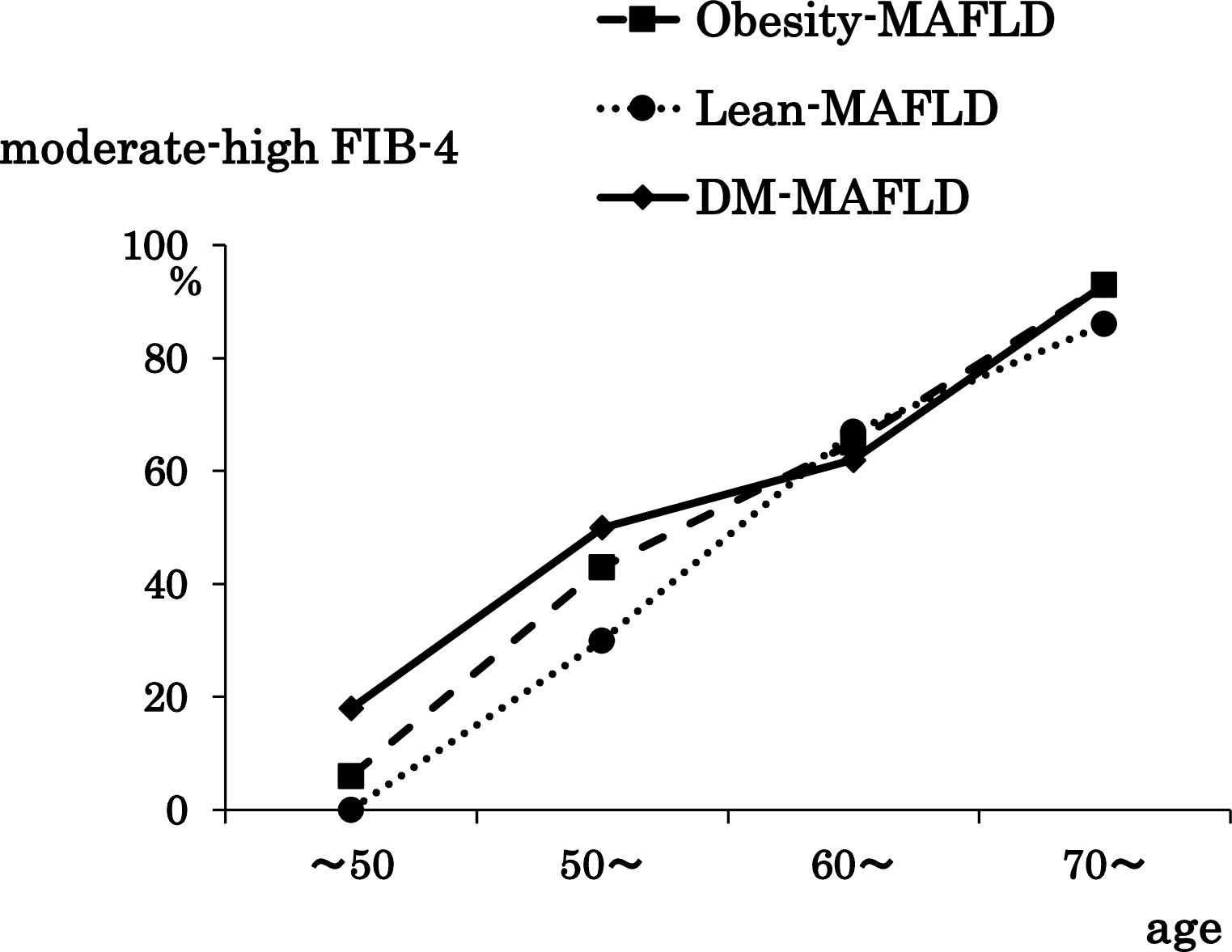

Supplement: S2 Fig — MAFLD, metabolic dysfunction-associated fatty liver disease; DM, diabetes mellitus; FIB-4, fibrosis-4. (TIF) [file pone.0269265.s003.tif]
