# Supplementary material for: A significant risk of metabolic dysfunction-associated fatty liver disease plus diabetes on subclinical atherosclerosis
Source: PLoS One. 2022 May 31;17(5):e0269265. doi: 10.1371/journal.pone.0269265 (PMC9154100; doi:10.1371/journal.pone.0269265)
Supplement: S1 Table — MAFLD, metabolic dysfunction-associated fatty liver disease; VAT, visceral adipose tissue; LDL-C, low-density lipoprotein cholesterol; HDL-C, high-density lipoprotein cholesterol; HOMA-R, homeostasis model assessment of insulin resistance; γGTP, gamma-glutamyl transferase; hs-CRP; high sensitive C-reactive protein; CACS, coronary artery calcification score; ba-PWV, brachial ankle pulse wave velocity; IMT, Intima media thickness. (DOCX) [file pone.0269265.s004.docx]

| **S1 Table. Clinical characteristic of MAFLD only and NAFLD** | | | | |
| --- | --- | --- | --- | --- |
| **Characteristics** | **MAFLD only**  134 (15.1) | **NAFLD**  268 (30.1) | ***P*** |  |
| Age (years) | 58.3 ± 10.7 | 60.2 ± 11.2 | 0.105 |  |
| Male | 123 (91.8) | 200 (74.6) | **0.000** |  |
| Body mass index (kg/m^2^) | 25.8 ± 3.7 | 25.6 ± 3.7 | 0.756 |  |
| VAT (cm^2^) | 140.7 ± 49.5 | 126.4 ± 53.8 | **0.010** |  |
| **Questioners** |  |  |  |  |
| Ever smoking | 98 (73.0) | 133 (49.6) | **0.000** |  |
| Exercise | 59 (44.0) | 86 (32.1) | **0.019** |  |
| Non-drinker | 4 (3.0) | 102 (38.1) | **0.000** |  |
| Hypertension | 76 (56.7) | 99 (39.6) | **0.001** |  |
| Diabetes mellitus | 45 (33.6) | 39 (15.6) | **0.000** |  |
| Dyslipidemia | 44 (32.8) | 95 (35.4) | 0.604 |  |
| **Blood test** |  |  |  |  |
| Total cholesterol (mg/dL) | 212.3 ± 37.3 | 198.8 ± 34.2 | **0.000** |  |
| LDL-C (mg/dL) | 119.9 ± 31.2 | 116.4 ± 29.3 | 0.269 |  |
| HDL-C (mg/dL) | 53.2 ± 12.5 | 49.9 ± 11.3 | **0.008** |  |
| Triglycerides (mg/dL) | 170.8 ± 116.3 | 131.3 ± 80.7 | **0.000** |  |
| Albumin (g/dl) | 4.37 ± 0.30 | 4.35 ± 0.28 | 0.531 |  |
| Platelet (x10^4^/ul) | 21.7 ± 4.9 | 22.6 ± 5.5 | 0.095 |  |
| Fasting blood sugar (mg/dL) | 117.8 ± 27.6 | 112.1 ± 20.4 | 0.019 |  |
| Hemoglobin A1c (%) | 5.92 ± 0.71 | 5.95 ± 0.65 | 0.709 |  |
| HOMA-R | 2.3 ± 1.9 | 2.4 ± 2.5 | 0.643 |  |
| Aspartate transaminase (U/L) | 29.1 ± 18.3 | 25.5 ± 9.8 | **0.010** |  |
| Alanine transaminase (U/L) | 30.2 ± 24.4 | 28.6 ± 15.2 | 0.432 |  |
| γGTP (U/L) | 77.1 ± 105.0 | 37.7 ± 27.6 | **0.000** |  |
| Elevated hs-CRP | 27 (20.1) | 48 (17.9) | 0.587 |  |
| **Subclinical atherosclerosis** |  |  |  |  |
| CACS > 0 | 63 (47.0) | 132 (49.3) | 0.672 |  |
| CACS > 100 | 29 (21.6) | 52 (19.4) | 0.598 |  |
| ba-PWV > 1400 (cm/s) | 74 (55.2) | 141 (52.6) | 0.567 |  |
| Carotid IMT ≥ 1.1 (mm) | 35 (26.1) | 92 (34.3) | 0.095 |  |

MAFLD, metabolic dysfunction-associated fatty liver disease; VAT, visceral adipose tissue; LDL-C, low-density lipoprotein cholesterol; HDL-C, high-density lipoprotein cholesterol; HOMA-R, homeostasis model assessment of insulin resistance;　γGTP, gamma-glutamyl transferase; hs-CRP; high sensitive C-reactive protein; CACS, coronary artery calcification score; ba-PWV, brachial ankle pulse wave velocity; IMT, Intima media thickness.
